# Paediatric Femur Fractures: A Plea for Minimally Invasive Surgery to Prevent Osteomyelitis

**DOI:** 10.5704/MOJ.1807.014

**Published:** 2018-07

**Authors:** N Mansor, AH Abdul-Rashid, S Ibrahim

**Affiliations:** Department of Orthopaedics and Traumatology, Universiti Kebangsaan Malaysia, Kuala Lumpur, Malaysia

Dear Editor,

We wish to highlight osteomyelitis occurring in two children after open reduction and plating of closed femur fractures. To the best of our knowledge, osteomyelitis following open reduction and plating of paediatric femur fractures has not been widely reported in the literature. To minimise the risk of developing osteomyelitis, we plead for minimally invasive surgery when treating paediatric femur fractures.

## Case 1

**Fig. 1:**
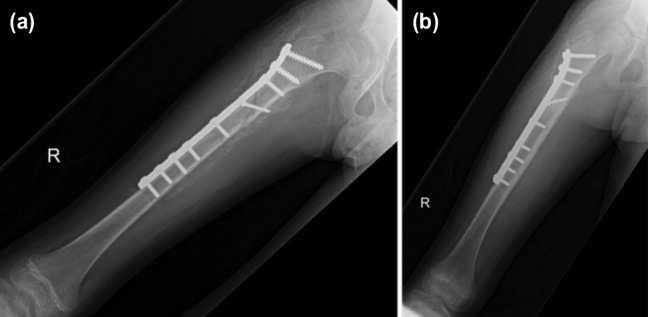
(a) The radiograph showing the involucrum in the proximal half of the femur. (b) The radiograph showing union of the fracture and resolution of infection 10 months after injury.

An 11-year old boy had sustained a closed comminuted fracture of the right femur after a motor vehicle accident. He had undergone open reduction and plating of the right femur two weeks after injury. He developed osteomyelitis (Fig. 1a) two weeks after surgery and was referred to our hospital for further management. Multiple debridements were required to treat the infection. The plate was not removed as it was not loose. Cultures grew Methicillin-Resistant Staphylococcus Aureus (MRSA), which was treated with rifampicin and fusidic acid orally for 6 weeks. His latest follow-up 10 months after injury showed healing of the fracture and resolution of the infection (Fig. 1b)

## Case 2

**Fig. 2:**
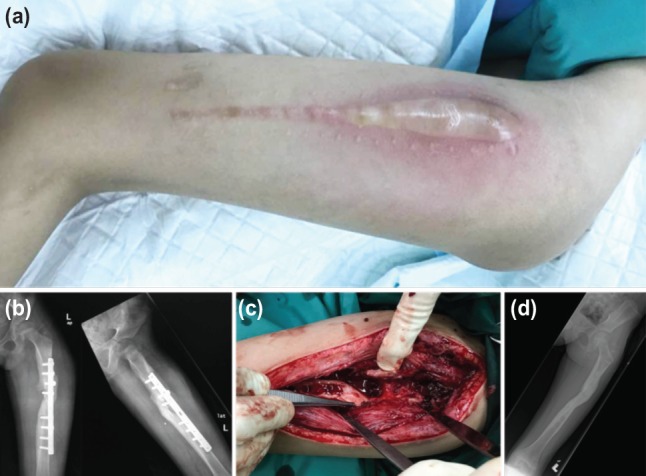
(a) A long surgical scar with a swollen and erythematous left thigh from osteomyelitis of the femur. (b) Radiograph showing osteomyelitis of the left femur and plate loosening. The fracture had united, (c) Intraoperative photograph after debridement and removal of plate. The femur (at the tip of the forceps) had united. (d) Radiograph one year after injury showing fracture remodelling and resolution of infection.

An 8-year old girl had sustained closed fractures of the left femur and ipsilateral clavicle following a motor-vehicle accident. She had undergone open reduction and plating of the left femur, while the clavicle fracture had been treated non-operatively. She was referred to our hospital five months after surgery with fever, pain and swelling over the surgical site (Fig. 2a). The plain radiograph showed osteomyelitis of the left femur and plate loosening (Fig. 2b). She was treated by debridement (Fig. 2b) and plate removal as the femur had united (Fig. 2c). The culture grew MRSA and she was treated with syrup trimethoprim/sulfamethoxazole for 6 weeks. The latest follow-up one year after injury showed the fracture had united and the infection resolved (Fig. 2d).

## Discussion

The surgical management of paediatric fractures has increased in recent years^1^. Helenius et al noted a 20 percent increase in the number of surgical procedures for treating paediatric fractures over a 10-year period^2^. However, the literature is sparse on osteomyelitis following open reduction and internal fixation of closed fractures in children.

May *et al* reported that three out of 85 children developed wound infection after plating of femur fractures. Eighty-nine percent had submuscular plating. No cases of osteomyelitis were recorded^3^.

Jolly *et al* compared the use of closed titanium nailing and open reduction and plating in femur fractures in children. Six out of 30 patients after plating developed wound complications including deep infection but osteomyelitis was not specifically listed^4^.

Khaled *et al* reported on 30 children with submuscular plate fixation of femur fractures. There were no cases of osteomyelitis^5^.

In conclusion, osteomyelitis in our two patients may have been prevented by using minimally invasive techniques. We recommend that pediatric femur fractures requiring surgery be treated using flexible nails, submuscular plating or external fixation instead of open reduction and plating.
